# Reconstruction of the DRUJ in a young adult after resection of a large exostosis of the distal radius

**DOI:** 10.1007/s11751-015-0224-4

**Published:** 2015-04-16

**Authors:** Bas R. J. Aerts, E. J. M. van Heeswijk, Annechien Beumer

**Affiliations:** Department of Orthopaedic Surgery, Upper Limb Unit, Amphia Hospital, Molengracht 21, 4818 CK Breda, The Netherlands

**Keywords:** Distal radioulnar joint, Forearm, Osteocartilaginous exostoses, Osteochondroma, MO, Reconstruction

## Abstract

The prevalence of known solitary exostosis is around 1–2 % in the general population. Treatment of an exostosis may consist of resection with or without further treatment for deformity. The distal radioulnar joint (DRUJ) acts as the link between radius and ulna at the wrist and is important in the transmission of load. Its anatomic integrity should be respected in surgical procedures or ulnar-sided wrist pain because of instability, limitation of forearm rotation and potential development of grip weakness may develop. We present a case of reconstruction of the DRUJ with distraction lengthening of the ulna after resection of a large exostosis of the distal radius that had resulted in a malformed and dysplastic ulna. This treatment in a young patient resulted in a stable, functional and congruent distal radioulnar joint.

## Introduction

An exostosis is a benign growth of bone. When exostoses are capped with cartilage, they are called osteocartilaginous exostoses (osteochondroma). The marrow of the exostosis is continuous with that of the underlying bone. An osteochondroma can appear solitary or as an autosomal dominant inheritance disease named multiple osteochondromas (MO). The disease mostly affects the long bones, pelvis and shoulder region. The prevalence of MO is around 1:50,000 and the incidence 1:18,000. The prevalence of known solitary exostosis is 1–2 % in the general population [1]. If radiologically more then two exostoses can be found in the epiphyseal region of long bones, the diagnoses MO is suspected [2]. However, the golden standard to diagnose MO is DNA testing, mutations in exostosin-1 (EXT1) and exostosin-2 (EXT2) genes leads to the diagnosis of MO in 95 % of the cases [3, 4].

Literature provides different numbers with respect to the affection of males versus females. According to some authors, penetrance is approximately 96 % in females and 100 % in males [5, 6]. According to others, it affects males more often then females (male-to-female ratio 1.5:1) [2, 7, 8]. The excess of males may be related to the fact that males have more frequent and severe complications of EXT [6, 9].

Patients with MO have a 2–4 % chance of developing a chondrosarcoma out of an exostosis; in solitary exostosis, this is around 1 % [6, 8, 10]. Deformities of the forearm can be found in approximately 30–60 % in patients with MO [11]. Treatment of the exostoses may consist of single resection of the exostosis with or without further treatment for deformity [12–17]. Exostoses of the distal forearm and their treatment may cause dysfunction of the stable distal radioulnar joint (DRUJ). The DRUJ acts as the link between radius and ulna and a pivot for pro- and supination. The joint is important in the transmission of load, and its anatomic integrity should be respected in surgical procedures if normal biomechanics are to be preserved [18]. DRUJ problems are responsible for ulnar-sided wrist complaints such as pain and weakness of grip, limitation of forearm rotation and instability. This article describes a case report of reconstruction of the DRUJ by osteotomy and distraction of a dysplastic distal ulna after resection of a large exostosis of the distal radius. The result was a pain-free forearm with a functional and stable DRUJ.

## Case report

**Fig. 1:**
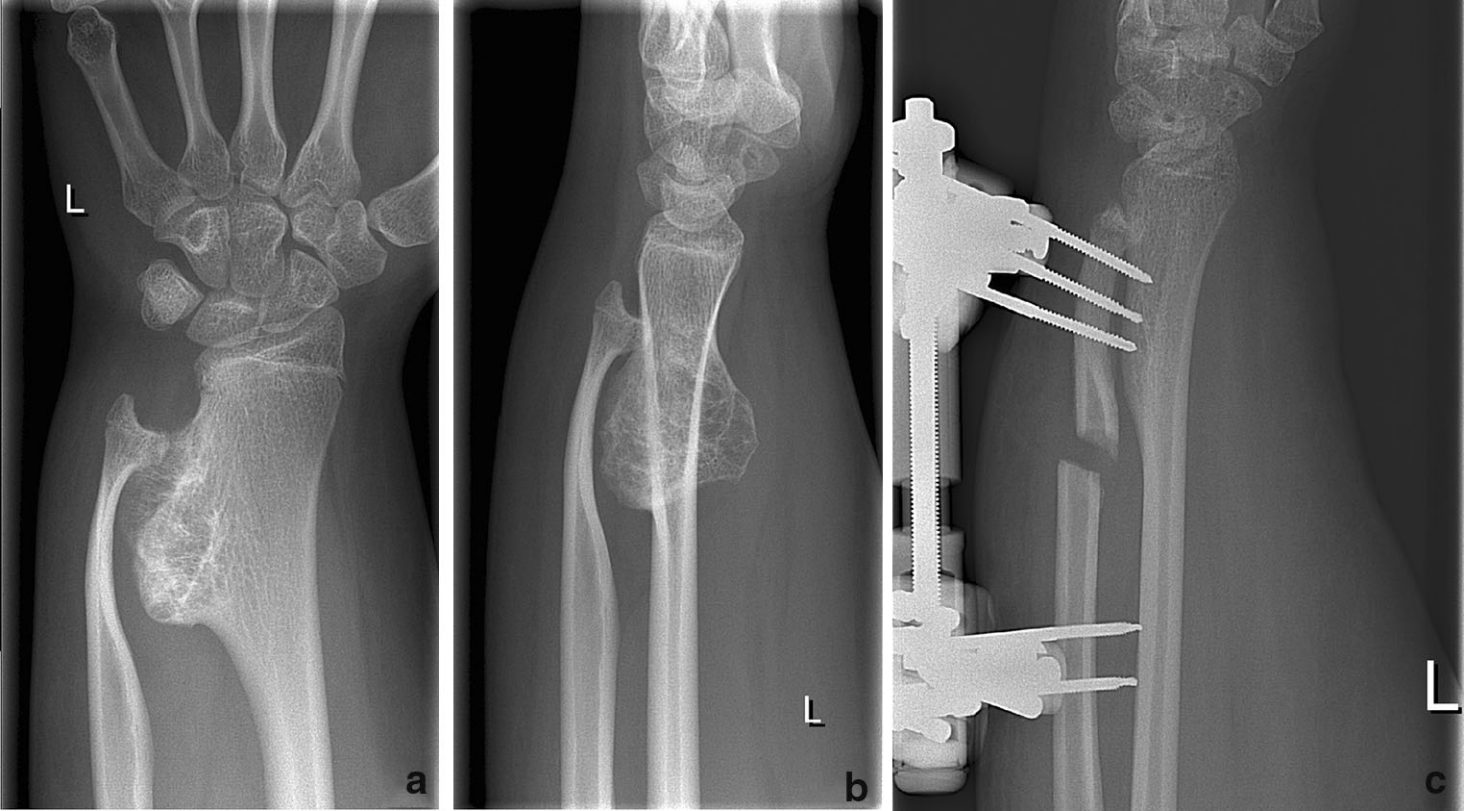
**a, b** Thin dysplastic ulna curved around exostosis. **c** Situation after the first procedure: external fixator with distraction device after osteotomy of the ulna

A 16-year-old healthy female, with a body mass index of 56, presented herself at the outpatient clinic of our hospital with a swelling of the left distal forearm that had developed slowly without a foregoing trauma. Her medical history did not reveal MO, nor did any family members suffer from MO. She visited our clinic because several close relatives suffered from other malignant diseases. She did not have any complaints at the first presentation, but a slightly limited rotation (pronation/supination of 60°/0°/45°). The overall Disabilities of the Arm, Shoulder and Hand (DASH) score was 7.5 [19]. There was no instability at the DRUJ or wrist. A bony swelling could be palpated at the dorso-ulnar side of the radius just proximal to the DRUJ. Radiography showed an exostosis at the ulnar side of the distal radius with reactive ulnar deformity. The ulna was thin and dysplastic due to usuration of the osteochondroma. The ulna curved around the exostosis with an ulna-14 mm configuration as a result (contralateral ulna-3 mm) (Fig. 1). Because of the benign appearance, minimal complaints and acceptable range of motion, it was decided not to perform invasive treatment at that time, but to check the lesion again in 6 months time. After 6 months, she suffered from progressive pain and her range of motion had decreased. No change in radiological appearance was found. For further evaluation, an MRI was made, which showed the exostosis with an overlaying cartilage cap without malignant characteristics. In addition, the MRI showed a relatively normal concave shape of the sigmoid notch of the radius, indicating that the joint had originally developed normal.

The case was presented to the Dutch Orthopedic Bone Tumor Society, which concluded that the most likely diagnosis would be osteocartilaginous exostosis. The commission advised surgical resection and, if possible, reconstruction of the distal radioulnar joint.

The first surgical procedure consisted of resection of the exostosis; a combined dorsal and volar approach was needed, because of the size of the exostosis. Then, osteotomy of the ulna was performed and the articulating surface of the ulna redirected towards the radial notch. An external fixator with distraction device was placed over the ulna in such a way that the displaced ulna would eventually grow into the sigmoid notch (Fig. 1).

Histology showed an osteochondroma, without malignant characteristics. Due to pain and swelling direct post-operatively, distraction of the ulna was not begun before 2.5 weeks after the first surgical procedure. Distraction was started with a rate of 0.5 mm per day for 2 weeks, but was reduced to 0.25 mm because of pain for the remaining period (when possible). During the distraction, the proximal ulna fragment deviated ulnarly resulting in a longer distraction time to reach the radial notch. In the distraction period, the patient developed a superficial infection at the external fixator pins. This infection was treated with oral antibiotics.

Three months after the first procedure, the ulna had covered 12 mm gap in length and the DRUJ appeared congruent; however, the distraction gap was not entirely filled with bone, indicating delayed union. Furthermore, the distracted ulna showed an angular deformity in the distracted area. Therefore, 3 months after the first procedure, the traction device was replaced by a static external fixator after reduction in the ulna. Post-operative treatment consisted of the use of a bone growth stimulator for 4 months. Only minimal signs of callus formation were seen after 4.5 months, and the patient experienced limitations in range of motion at her work as kindergarten teacher because of the external fixator. Therefore, the external fixator was removed, and plate osteosynthesis combined with bone grafting was performed. The defect was filled with autologous bone harvested from the crista iliaca bone combined with a demineralised bone graft system. (BONUS II DBM Matrix from Biomet Biologics Inc, Warsaw, IN, USA.)

**Fig. 2:**
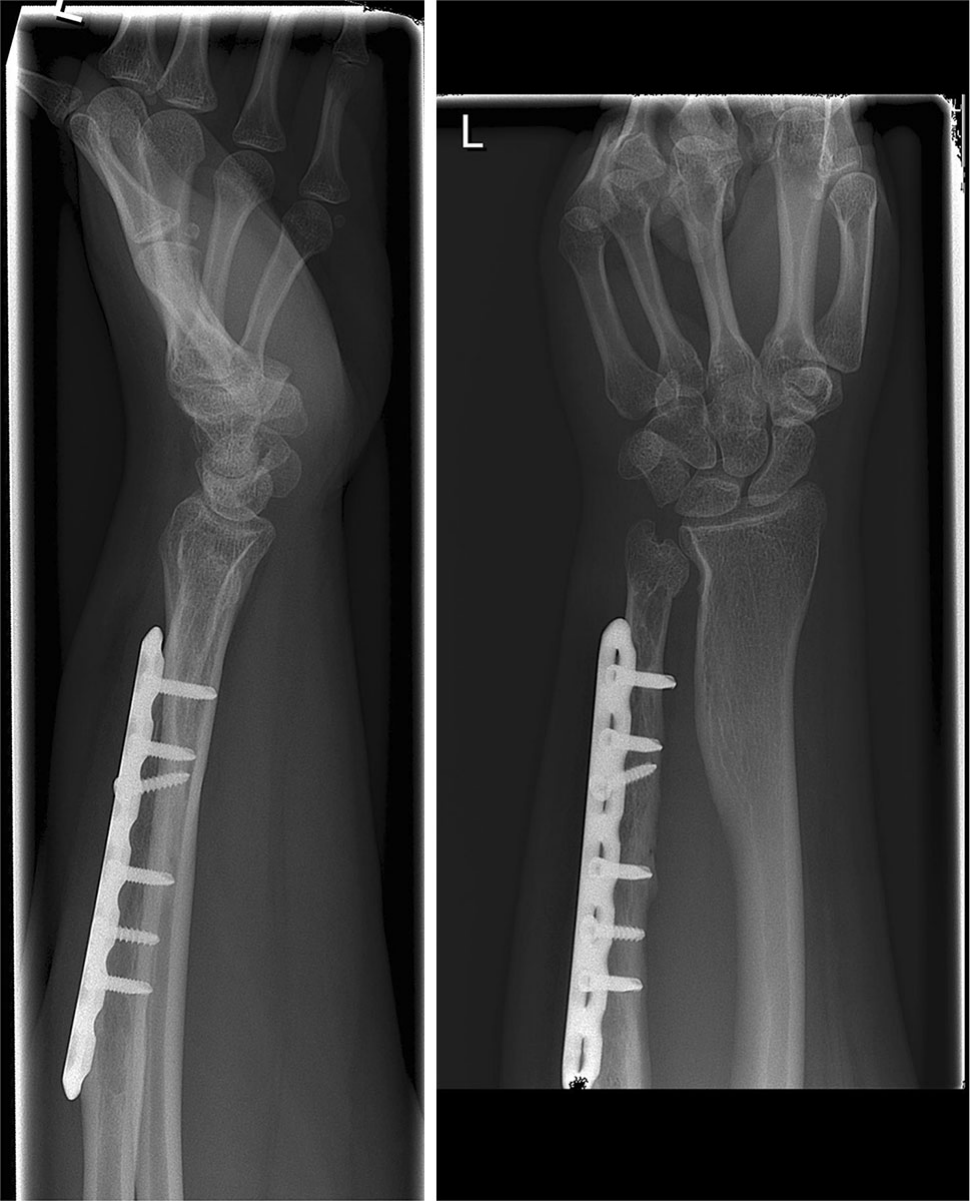
Situation 42 months after the last procedure: a congruent DRUJ

Clinical and radiological consolidation was achieved at 27 months after the first procedure. At final follow-up, 51 months after the first procedure, patient was very satisfied and the positive result of this procedure had encouraged her to have bariatric surgery. Her BMI had dropped from 56 to 30 resulting in slightly irritation of the plate. The VAS for pain score was 0. Clinical investigation revealed an excellent range of motion with flexion/extension 80°/0°/80° and pronation/supination 80°/0°/85°. The DRUJ was stable, and the grip strength of the wrist was equal to the controlateral wrist (right: 28 kg and left: 26 kg). Radiographs showed a normal sized ulna and a congruent DRUJ (Fig. 2). The overall DASH score was 7.5 and when corrected for her job 6.6. She had resumed her studies and resumed her work as kindergarten teacher and clothes store co-worker. Because of the slight irritation, it was decided to remove the plate in the near future.

## Discussion

According to our knowledge, this is one of the first reports of reconstruction of the DRUJ resulting in a nearly normal joint in a young adult with closed epiphyses by distraction lengthening of a dysplastic distal ulna. After resection of a large exostosis, we reconstructed the DRUJ by reduction and distraction lengthening of the dysplastic and deformed distal ulna. The performed procedures resulted in a stable DRUJ with grip strength equal to the controlateral wrist.

It can be assumed that the triangular fibrocartilage complex (TFCC) does not have completely normal anatomy. Nevertheless, the DRUJ was stable after distraction and reconstruction. We assume most of the stability is a result of an intact interosseous membrane and ligament. The pre-operative examination showed a stable connection between radius and ulna with the distal interosseous membrane (DIOM) clearly visible just proximal to the exostosis on the pre-operative MRI. Furthermore, fibres of an intact and elongated TFCC could be seen in an aberrant form curling around the exostosis. Both structures, together with the proximal radioulnar joint (PRUJ), play an important role in the distributing of applied load of the forearm and were carefully respected during the surgical procedures. In this case, the presence of those structures in combination with the long distraction and consolidation time will have led to the clinically stable DRUJ [20, 21].

Therefore, it might be important for other surgeons to analyse and respect these structures and to distract slowly in similar cases.

In this case, a delayed union was observed. Several factors might have contributed to this. Delayed union is not uncommon after distraction lengthening. Several authors described delayed and non-unions after distraction lengthening. Peterson et al. [22] describes three delayed unions are described in 13 children who underwent distraction lengthening of the ulna, and Taghinia et al. [23] describe three non-unions and one delayed union in eleven patients who underwent two-stage distraction lengthening of the forearm. The external fixator that was placed during the first procedure was placed in such an angle that the distal ulna was redirected to the radial notch. Although clinically stable, it might have allowed some rotational forces. Furthermore, the distracted ulna was very thin and dysplastic with hardly any spongious bone at the start of distraction. Other causes of delayed consolidation may occur secondary to patient factors including infection, malnutrition or metabolic diseases. Possible underlying causes might be the fact that there was a dystrophic ulna and almost no bone marrow of the cavity of the ulna was present. Also the superficial infection of the external fixator pins can influence the delayed union. In retrospection, one could debate whether plate fixation should have been performed earlier in our case because it would have resulted in earlier consolidation [24].

We chose to reconstruct the DRUJ after resection of the exostosis by osteotomy and distraction lengthening of the ulna. Other treatment options could have been distal ulna resection (Darrach’s procedure), distal radioulnar arthrodesis with intentional distal ulnar pseudoarthrosis (Sauvé & Kapandji procedure) or prosthetic replacement of the distal ulna. The Sauvé-Kapandji procedure provides support for the ulnar carpus but would have resulted in instability problems of the ulna with radioulnar impingement. The same can be seen after Darrach’s procedure, especially in young high-demanding patients [25]. Prosthetic replacement would have been difficult with the deformed ulna with a very thin shaft and has additional risks, including infection and prosthetic loosening. Furthermore, a joint prosthesis would not be advised in such a young patient. Although these procedures can be used as a salvage to treat the remaining deformity after resection of the exostosis, most procedures would not be advised in a young patient and would need a certain amount of ‘normal’ anatomy (i.e. congruent joint with intact stability to start with). This was not the case in this patient, nor was the stability of the distal ulna after resection of the exostosis good enough to be left untreated. It is uncertain what the long term results of this reconstruction will be, but other treatment options mentioned will be available if the patient would develop complaints such as osteoarthritis in the future. We are aware of the limitations of this study since we have only one case with a relatively short follow-up.

The same sort of procedure has been described in six patients with dislocated radial heads because of MO; in these cases, shortening osteotomy of the radius followed by lengthening of the ulna in case of longstanding radial head dislocation was needed to realign the joint [26]. In these patients, the technique described resulted in satisfactory functional and cosmetic results.

In older patients, however, other treatment options might have been preferred as primary surgery. Resection of the exostosis and placement of ulnar head or (constrained) DRUJ prosthesis would have resulted in significantly shorter duration of treatment.

In conclusion, this case report shows that osteotomy and distraction lengthening of the dislocated ulna in a young patient might result in a stable, functional and congruent DRUJ.

The technique described is also interesting to treat other dislocated or dysplastic joints in growing individuals.
